# Direct Bioelectricity Generation from Sago Hampas by *Clostridium beijerinckii* SR1 Using Microbial Fuel Cell

**DOI:** 10.3390/molecules24132397

**Published:** 2019-06-28

**Authors:** Mohd Azwan Jenol, Mohamad Faizal Ibrahim, Ezyana Kamal Bahrin, Seung Wook Kim, Suraini Abd-Aziz

**Affiliations:** 1 Department of Bioprocess Technology, Faculty of Biotechnology and Biomolecular Sciences, Universiti Putra Malaysia, 43400 UPM Serdang, Malaysia; 2 Department of Chemical and Biological Engineering, Korea University, Seoul 136-701, Korea; 3 Department of Chemistry, Faculty of Science and Technology, Universitas Airlangga, Surabaya 60115, Indonesia

**Keywords:** sago hampas, starch, bioelectricity generation, *Clostridium beijerinckii*, microbial fuel cell

## Abstract

Microbial fuel cells offer a technology for simultaneous biomass degradation and biological electricity generation. Microbial fuel cells have the ability to utilize a wide range of biomass including carbohydrates, such as starch. Sago hampas is a starchy biomass that has 58% starch content. With this significant amount of starch content in the sago hampas, it has a high potential to be utilized as a carbon source for the bioelectricity generation using microbial fuel cells by *Clostridium beijerinckii* SR1. The maximum power density obtained from 20 g/L of sago hampas was 73.8 mW/cm^2^ with stable cell voltage output of 211.7 mV. The total substrate consumed was 95.1% with the respect of 10.7% coulombic efficiency. The results obtained were almost comparable to the sago hampas hydrolysate with the maximum power density 56.5 mW/cm^2^. These results demonstrate the feasibility of solid biomass to be utilized for the power generation in fuel cells as well as high substrate degradation efficiency. Thus, this approach provides a promising way to exploit sago hampas for bioenergy generation.

## 1. Introduction

Bio-electrochemical systems (BESs) are providing the innovative technologies that utilize the biological redox catalytic activity with the combination of abiotic electrochemical reactions [1,2], which are normally classified based on their applications, such as the generation of energy [3], chemicals and water treatment processes [4,5]. In fact, BESs used for energy generation are known as bio-electrochemical fuel cells, which can be further divided into enzymatic fuel cells (EFCs) and microbial fuel cells (MFCs). Microbial fuel cells (MFCs) represent a new platform of technology that converts the chemical energy in biomass into bioelectricity through metabolic activity of electrochemically active bacteria attached to the electrode [6]. Hence, the exploitation of bacteria as the electrocatalysts in MFCs has given the capability to directly generate electricity from a various type of substrate, including organic acids (acetate, butyrate, lactate), fermentable sugars (glucose, xylose) and carbohydrates (sucrose, starch) [7].

In spite of that, there is still more improvement required for reactor configurations and electrolyte design in MFCs, in order to enhance the effectiveness in terms of productivity as well as production cost [1]. According to Liu et al. [8], the substrate is indeed one of the most important biological factors affecting the overall performance of microbial fuel cells, including bioelectricity generation and operational cost. On the other hand, due to the versatility of fuel used in MFC, a novel approach has been introduced for MFCs as bioenergy production and biomass degradation technologies.

The utilization of biomass for energy generation has drawn considerable attention due to their abundance and ready availability [9]. In review, there are few sources of biomass that have been studied for the generation of bioelectricity, including potato waste [10], rice straw [11], wheat straw [12], and corn stover [13,14]. Du and Li [10] demonstrated bioelectricity generation in terms of current density from a mixture of cooked and uncooked potato, of which the maximum current density obtained was enhanced to 278 mA/m^2^/d (10 fold increment). Hassan et al. [11] reported that the power density obtained from 1 g/L of initial rice straw concentration was 143 mW/m^2^ with the coulombic efficiency (CE) in the range of 45.3–54.3%. In addition, a 123 mW/m^2^ of power density was obtained by using a wheat straw hydrolysate, with the CE in the range of 15.5%–37.1% [12]. These findings provide evidence of the promising platform that utilizes the readily available biomass for bioelectricity generation. However, the use of other biomass should be investigated in order to provide a better understanding of and optimizing the generation of bioelectricity in MFCs.

In Malaysia, Sarawak is known as one of the largest sago starch exporters in the world, accounting for more than 25,000 mt/year of sago starch [15]. This figure is expected to increase by 15% to 20% every year [16]. It should be noted that the increase in a number of products will significantly increase the amount of waste produced from the sago processing mill, which subsequently is associated with environmental pollution problems if it is poorly handled. Sago hampas is a starchy lignocellulosic biomass produced during the extraction of sago starch which contains 58% starch, 23% cellulose, 9.2% hemicellulose and 3.9% lignin [17,18,19]. This sago hampas has a great potential to be utilized as a feedstock for bioenergy production. In MFCs, a starch component has been used for the generation of bioelectricity. Niessen et al. [20] has demonstrated the exploitation of *Clostridium beijerinckii* and *Clostridium butyricum* in bioelectricity generation using starch as an electron donor. The aforementioned authors reported that the current density obtained from *C. butyricum* and *C. beijerinckii* was 1.3 mA and 0.8–1.0 mA, respectively. On the other hand, the starch-based wastewater has been utilized as a fuel in MFCs, generating the highest voltage output of 281 with the power density of 139.8 mW/m^2^ [21].

Therefore, this paper has aimed to utilize sago hampas as a substrate in the direct generation of bioelectricity by *C. beijerinckii* SR1 using a microbial fuel cell. Subsequently, the performance of sago hampas will be compared to the hydrolyzed sago hampas.

## 2. Results and Discussion

### 2.1. Characterization of Sago Hampas

In MFCs, the substrate is the most important fermentation factors affecting the production cost as well as bioelectricity generation [8]. Sago hampas is a great potential biomass produced after the extraction of starch and contains a significant amount of starch materials and fiber [17]. Table 1 illustrates the chemical composition of sago hampas used in this study for the generation of bioelectricity.

Sago hampas is a great potential biomass that consists both starch content as well as lignocellulosic materials. All the values (Table 1) except starch are almost comparable to the previous reports [17,18]. It is found that the amount of starch used in this study (58% on a dry basis) is slightly higher as compared to the previous study (30–50% on a dry basis). These differences in starch value are greatly dependent on the extraction protocol applied in the mills. Awg-Adeni et al. [17] have explained that higher amount of starch will be found in the sago hampas mainly due to the food grade starch demand, thus the factory has reduced the recycling process in the extraction to ensure the starch whiteness. It should be noted that this study was aimed to utilize the starch content in the biomass for the generation of bioelectricity.

### 2.2. Preliminary Experiment: Bioelectricity Generation from Commercial Starch

**Figure 1:**
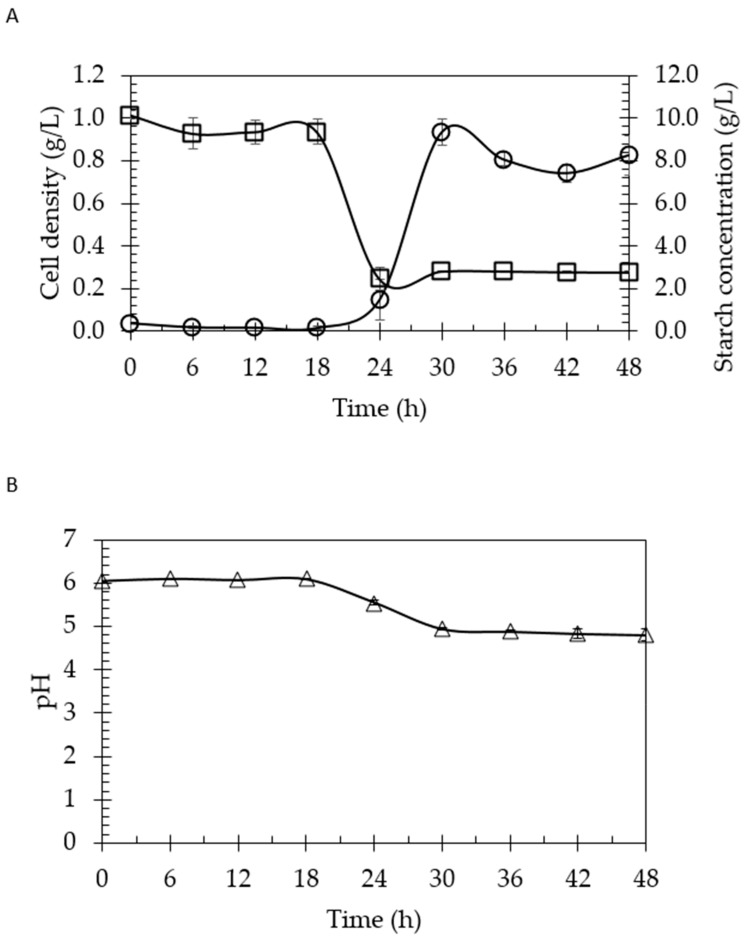
Profiling of *Clostridium beijerinckii* SR1 in 10 g/L of commercial starch. (**A**) The cell density (○) and commercial starch consumption (□) as a function of time. (**B**) pH changes.

In this study, *Clostridium beijerinckii* SR1 was employed as a biocatalyst due to its high productivity and efficiency as a biocatalyst for hydrogen, which is the major electron donor in bioelectricity generation [20]. On the other hand, this bacterial strain is able to digest vast types of substrate, including low molecular compound like organic acids (acetate, butyrate), monosaccharides like glucose, and even starch [20,22,23]. It is also reported that *Clostridium* sp. have the ability to express α-amylase and glucoamylase, which are the important enzymes in degrading the starch. Figure 1 shows the growth profiling of *Clostridium beijerinckii* SR1.

Based on Figure 1, the highest cell density obtained was 0.93 ± 0.05 g/L at 30 h of fermentation. The pH dropped below pH 5 after 30 h, indicated by the acid production of this strain in the acidogenesis process. This situation caused the suppression of this bacteria to further grow when the accumulation of acids in the fermentation broth reached the threshold value just before the cells began to enter the stationary phase [24].

**Figure 2:**
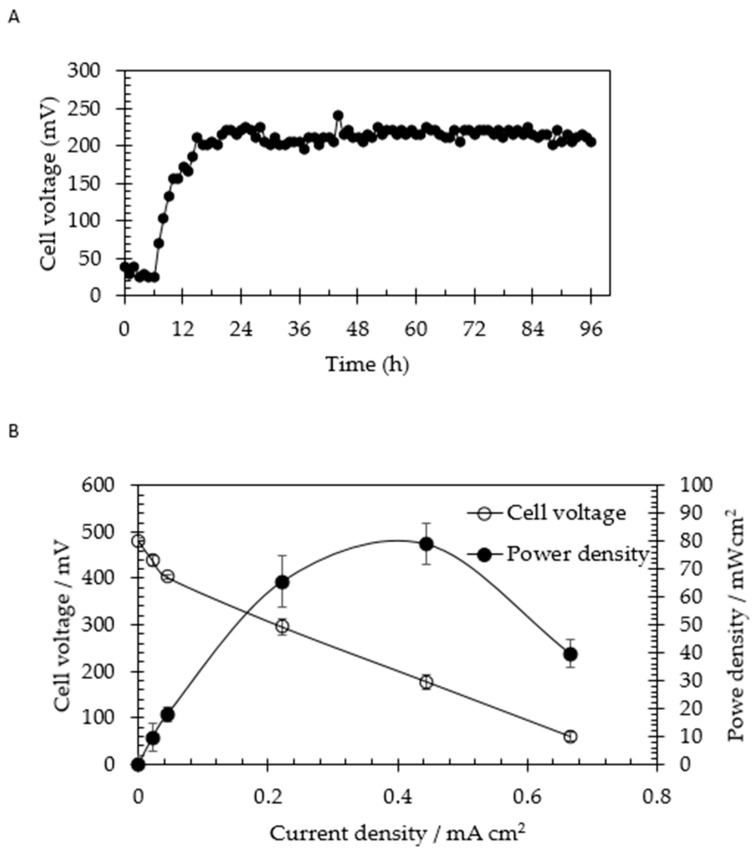
Bioelectricity generation from commercial starch by *Clostridium beijerinckii* SR1. (**A**) Voltage generation from 10 g/L of starch by *C. beijerinckii* SR1 as a function of time. (**B**) Power density and cell voltage generation as a function of current density.

A preliminary experiment was conducted using commercial starch as an electron donor to demonstrate the capability of *C. beijerinckii* SR1 to generate the bioelectricity in MFCs system. Figure 2 illustrates the bioelectricity generation powered by the commercial starch as an electron donor by *C. beijerinckii* SR1 as a biocatalyst. Initially, when starch was introduced into the system, the circuit voltage (open circuit voltage (OVC)) of 26.4 ± 6.4 mV (0–6 h) was generated immediately and this might be due to the difference between anodic and cathodic potential of the two electrodes. Min et al. [25] explained that this situation might be due to the biological and chemical factors. Thereafter, the cell voltage (OVC) was increased and stabilized to 214.2 ± 7.3 mV (7–96 h) due to the biological activity of *C. beijerinckii* SR1. The voltage generation using two chambers of MFCs in the study was similar to the data showed by Min et al. [25].

The maximum power density obtained from the polarization curve (Figure 2B) was 78.92 ± 7.48 mW/cm^2^ (at an applied current of 1.0 mA). The results showed that *C. beijerinckii* SR1 is a great potential biocatalyst capable of generating bioelectricity powered by starch. This situation is explained by the catalytic oxidation of hydrogen [20]. *Clostridium* sp. is a well-known biocatalyst that synthesizes biohydrogen via a ferredoxin-linked pathway of the butyric acid fermentation, then produces two moles of hydrogen per glucose unit in the fermentation of starch [26,27,28]. As explained by Niessen et al. [20], the main criteria of the selection of biocatalyst in the fuel cell are based on the efficiency of hydrogen synthesis as well as the ability to feed on the desired carbon source. In this study, we demonstrated the ability of *C. beijerinckii* SR1 in the generation of bioelectricity as well as the utilization of a macromolecular natural substrate (starch) and further investigated with solid biomass, which is sago hampas.

### 2.3. Direct Bioelectricity Generation by Clostridium beijerinckii SR1 Using Sago Hampas

**Figure 3:**
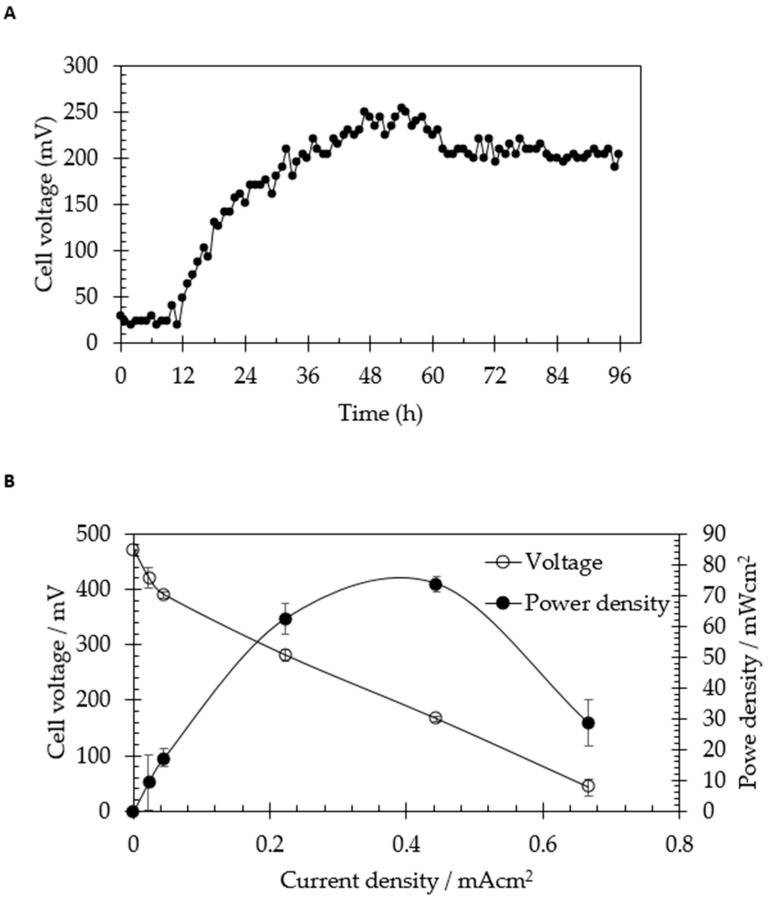
Bioelectricity generation from sago hampas by *Clostridium beijerinckii* SR1. (**A**) Voltage generation from 20 g/L of sago hampas by *C. beijerinckii* SR1 as a function of time. (**B**) Power density and cell voltage generation as a function of current density.

Figure 3 exemplifies the bioelectricity generation as a function of cell voltage and power density by *C. beijerinckii* SR1 fueled by sago hampas. When the sago hampas was introduced, 33.8 ± 5.5 mV (0–11 h) of voltage output was generated out of the gate. Subsequently, the cell voltage was increased and stabilized at 211.7 ± 2.3 mV (12–96 h). The maximum power density recovered was 73.78 ± 2.6 mW/cm^2^, at applied current 1.0 mA. The results obtained demonstrated that the performance of bioelectricity generation from sago hampas was almost comparable to commercial starch. This situation explained the potential of sago hampas as a replacement of commercial starch in bioelectricity generation using MFCs. The cell voltage output obtained in this study was slightly lower as compared to study made by Ahmed et al. [21]. The aforementioned authors used the starch-based wastewater as an electrolyte in a single chamber MFC with the maximum power density of 139.8 mW/m^2^ and chemical oxygen demand (COD) removal of 86.4%. On the other hand, Neissen et al. [20] obtained 1.86 mW/cm^2^ of power density by *C. butyricum* from 10 g/L of starch.

**Figure 4:**
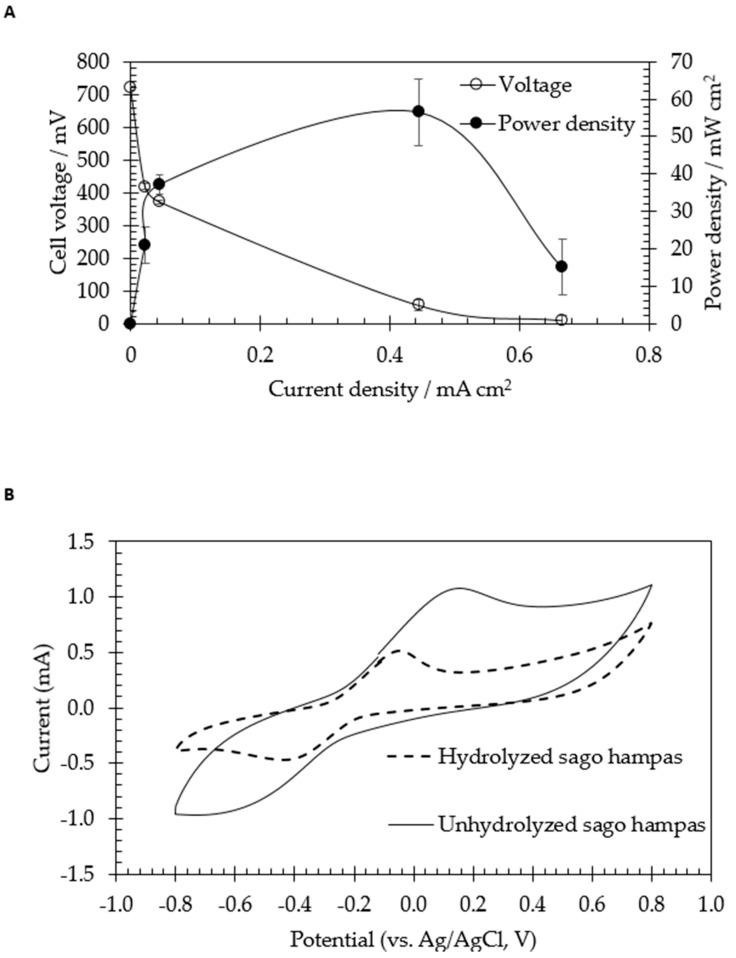
Generation from sago hampas hydrolysate by *Clostridium beijerinckii* SR1. (**A**) Power density and cell voltage generated from 10 g/L of glucose content in sago hampas hydrolysate as a function of current density. (**B**) Cyclic voltammetry profile of *Clostridium beijerinckii* SR1 recorded at 96 h after the start of anaerobic fermentation using unhydrolyzed and hydrolyzed sago hampas as substrate. The scan rate was 10 mV/s.

On the other hand, the result obtained from sago hampas was compared with the bioelectricity generation powered by hydrolyzed sago hampas, also known as sago hampas hydrolysate. Figure 4a illustrates the bioelectricity generation in the freshly inoculated anaerobic culture of *C. beijerinckii* SR1 containing sago hampas hydrolysate as a substrate. The concentration of 10 g/L of glucose content in the sago hampas hydrolysate was subjected as an initial carbon source. The maximum power density obtained from hydrolyzed sago hampas was 56.5 mW/cm^2^, which was slightly lower than unhydrolyzed sago hampas. Based on the result obtained, it is suggested that there is no additional step (hydrolysis process) required in order to utilize the sago hampas as a substrate for the generation of bioelectricity.

Based on the result obtained, the current obtained was 2.4 mA. The electrochemical behavior of MFC using sago hampas by *C. beijerinckii* SR1 was further evaluated using cyclic voltammetry (CV) that measures the potential differences across the interface as well as the redox of the component involved of the biochemical system. Figure 4b shows the redox potential signal obtained from hydrolyzed sago hampas and unhydrolyzed sago hampas. Based on the data obtained, unhydrolyzed sago hampas emitted a higher oxidation potential peak compared to hydrolyzed sago hampas, with the potential of 0.15 V and −0.02 V, respectively. The voltammogram shape obtained in this study was in agreement with Finch et al. [29]. The results obtained suggested that the electrochemical activity of *C. beijerinkcii* SR1 might be due to the cell surface cytochrome(s) [29,30,31]. *C. beijerinckii* SR1 showed quasi-reversible redox reaction in CV with a sharper reduction peak as compared to the oxidation peak. It also appears that there was more efficient electron flow towards the bacteria cells.

Based on experimental results obtained, the performance of *C. beijerinckii* SR1 on the bioelectricity generation from commercial starch and sago hampas was subjected to the assessment of coulombic efficiency (CE). The CE was a function of either external circuit resistance or substrate concentration. The total starch consumed from sago hampas was 95.1% corresponding to a CE of 10.7%. This indicates that the major electron produced resulted from the degradation of starch and was not fully associated with power generation, but instead correlated to biomass production as well as fermentation products. *Clostridium* were known to be solvents (acetone–butanol–ethanol) and organic acids (acetic acid and butyric acid) producers. In this study, it is determined that this particular strain produced 4.02 ± 0.52 g/L of total solvents and 6.71 ± 0.45 g/L of total organic acids. This situation was in agreement with Lu et al. [32], which explained that the lower CE in bioelectricity generated by bacteria could be caused from the correlation of electron acceptor diffusion as well as other processes, including biomass production and fermentation. Logan et al. [33] have reported that the CE was diminished by several factors, including the competitive processes for the production of by-products as well as bacterial growth.

## 3. Materials and Methods

### 3.1. Substrate Collection and Preparation

The biomass used was sago hampas, which was collected from River Link Sago Resources Sendirian Berhad Company (Sdn. Bhd.), Mukah, Sarawak, Malaysia. Sago hampas was dried by using an oven dryer at 65 °C overnight. The dried sago hampas was then kept in the sealed plastic bags and stored at room temperature for further use.

### 3.2. Hydrolysis of Sago Hampas

Sago hampas containing 58% starch (dry basis) was subjected to the hydrolysis process [17,18]. A suspension of 7% (*w*/*v*) of sago hampas was boiled in 0.1 M KH_2_PO_4_ buffer solution (pH 4) for 15 min and subsequently cooled down to 60 °C at room temperature. A 5.56 U/mL of dextrozyme (Novozymes, Bagsværd, Denmark) with glucoamylase activity of 195.3 U/mL was added into the suspension. The mixture was incubated at 60 °C for 60 min in a water bath for the hydrolysis process. The mixture was continuously stirred to ensure the homogeneity of substrate end enzyme throughout the process. Then, the suspension was submerged into an ice-water bath to prevent further hydrolysis. The hydrolysate was then separated from the lignocellulosic fiber residual by using a filtration (100 mesh) and centrifugation (12,000 rpm for 15 min) technique. The hydrolysate obtained, referred as sago hampas hydrolysate was subjected to analytical procedures for its reducing sugars and glucose content. The hydrolysis yield (%) was calculated as:

Glucose produced from sago hampas (g)/Dry sago hampas (g) × 100.(1)

### 3.3. Bacterial Strain and Medium

The single culture, *Clostridium beijerinckii* SR1 employed was obtained from Madihah’s laboratory culture collection, Universiti Teknologi Malaysia [34]. Aliquots of 1 mL of the stock culture was inoculated into 125 mL serum bottle, with 100 mL working volume of oxygen free and sterilized growth medium, pH 5.5. The medium [20] contained 10 g substrate (sago hampas), 5 g yeast extract, 10 g peptone, 0.5 g l-cysteine-HCl, 0.4 g NaHCO_3_, 8 mg CaCl_2_, 8 mg MgSO_4_, 40 mg KHSO_4_, 80 mg NaCl and 1 mg resazurin (per L). The pH was adjusted using 1.0 M of NaOH and HCl. The medium was purged with nitrogen gas for 15 min, and autoclaved at 121 °C for 15 min. It was incubated at 120 rpm and 37 °C for 24 h in a shaker incubator (Labwit, Victoria, Australia). The inoculum was freshly prepared for every bioelectricity generation in microbial fuel cells (MFCs).

### 3.4. MFC Construction and Operation

The MFCs used was the H-type, which consisted of conjoint two chambers; anode and cathode. An anaerobic chamber (anode) and aerobic chamber (cathode) were separated by the proton exchange membrane (PEM) (Nafion N117, Fuel Cell Earth, Massachusetts, United State of America). The electrodes (1.5 × 1.5 cm) used in the anode and cathode compartment were the carbon cloth (5% wetproofing) (Fuel Cell Earth, Massachusetts, USA) and 20% platinum on Vulcan—carbon cloth (Fuel Cell Store, USA), respectively. The pretreatment of electrodes and electrical connection were done based on that previously described by Oh et al. [35].

The MFCs were operated at a fixed external circuit resistance (300 Ω). Both chambers were filled with 200 mL working volume of fermentation medium. Sago hampas was added as an electron donor (commercial starch is used as a control) and 10% (*v*/*v*) of *C. beijerinckii* SR1 was inoculated into the anode compartment contained in the fermentation medium prior to the fermentation, simultaneously. Then, the nitrogen gas was purged into the anode compartment for 15 min to prepare the anaerobic condition. In the cathode compartment, 50 mM K_3_Fe(CN)_6_ was used as the terminal electron acceptor.

### 3.5. Calculation and Chemical Analysis

All the analysis samples were run for triplicates. Starch content was determined using the method explained by Nakamura [36]. The glucose concentration was determined by using high performance liquid chromatography (HPLC) (Jasco, Tokyo, Japan) equipped with Refractive Index (RI) detector and Rezex RCM-Monosaccharide (Reliability-Centered Maintenance) column (Phenomenex, USA) at 80 °C with deionized water as a mobile phase at flow rate of 0.6 mL/min. The solvent produced was analyzed using Gas chromatography (Shimazu, GC-17A, Kyoto, Japan) occupied with column BP-21 with helium as a carrier gas. Cell voltages (V) were measured across a fixed external circuit resistor (300 Ω) using a data acquisition system (LabJack U12 Series, LabJack Corporation, US) that recorded every 10 min. The polarization curve was determined by Autolab PGSTAT204 (Metrohm, Malaysia) imposing varying current (0–1.5 mA). The electron discharge pattern was also evaluated using Autolab PGSTAT204 (Metrohm, Malaysia) by studying the cyclic voltammetry (CV). CV was evaluated over a voltage range of −0.8 mV to + 0.8 mV at the scan rate of 10 mV/s. An Ag/AgCl electrode was used as a reference electrode, while an anode electrode and cathode electrode acted as a working and counter electrode, respectively. Power (P) density was calculated as:

P = I × V (I = V/R)(2)

where, I (A) is the current, V (V) is the voltage and R (Ω) is the external resistance. The power was normalized to the anode surface area [35,37]. The electrical charge (in coulombs) was calculated by integrating the current over time and the amount of substrate removal during a MFC operation [25,35,38]. Starch content was measured using iodine starch colorimetric explained by Nakamura [36].

The coulombic efficiency (CE) was calculated based on the total coulombs calculated by integrating the current over time (CP) and the theoretical amount of coulombs available from COD (CT) using Equation (3) as [32]:

CE = CP/CT × 100%.(3)

The current over time (CP) is defined as CP = ∫ I dt, whereas the theoretical amount of coulombs (CT) is defined as CT = FbV ∆COD/M, where F is the Faraday’s constant (96485 C/mol of electrons), b is the number of moles of electrons produced per mol of substrates (b = 4), V (L) is the liquid volume, ∆COD (g/L) is the concentration difference, and M is the molecular weight of substrate (M = 23).

## 4. Conclusions

This study evaluated the capability of the MFCs system in utilizing the solid biomass, sago hampas for the generation of bioelectricity by single culture, *Clostridium beijerinckii* SR1 in order to be one-step closer to better understanding of MFCs concept. In this study, three sources of substrate were tested, including commercial starch, unhydrolyzed and hydrolyzed sago hampas. The maximum power density generated by *C. beijerinckii* SR1 from unhydrolyzed sago hampas is 73.78 mW/cm^2^ which is a comparable performance compared to commercial starch (78.92 mW/cm^2^), while hydrolyzed sago hampas gave lower power density (56.5 mW/cm^2^). These results indicate the value of unhydrolyzed sago hampas in being utilized directly as a carbon source without any further hydrolysis process for the generation of bioelectricity. It can be concluded that sago hampas has shown a great potential replacement for commercial starch in bioelectricity generation using MFCs. The improvement in terms of reactor configuration as well as electrodes manipulation could further enhance the generation of bioelectricity by using sago hampas as a feedstock in MFCs.

## Figures and Tables

**Table 1:** Chemical composition of sago hampas.

Chemical Compositions (%)	This Study	Reference [17]	Reference [18]
Starch	58 ± 0.02	30–45	49.5
Cellulose	21	n.d	26
Hemicellulose	13.4	n.d	14.5
Lignin	5.4	n.d	7.5
Moisture	4.7 ± 0.42	5–7	n.d
Ash	3.13 ± 0.13	3–4	n.d
pH	4.49 ± 0.1	4.6–4.7	n.d

%—percentage in dry basis, n.d—not determined.
